# GenomeGraphs: integrated genomic data visualization with R

**DOI:** 10.1186/1471-2105-10-2

**Published:** 2009-01-06

**Authors:** Steffen Durinck, James Bullard, Paul T Spellman, Sandrine Dudoit

**Affiliations:** 1 Life Sciences Department, Lawrence Berkeley National Laboratory, 1 Cyclotron Rd, Berkeley, CA 94720, USA; 2 Division of Biostatistics, School of Public Health, UC Berkeley, 101 Haviland Hall, Berkeley, CA 94720-7358, USA; 3 Department of Statistics, UC Berkeley, 367 Evans Hall, Berkeley, CA 94720-3860, USA

## Abstract

**Background:**

Biological studies involve a growing number of distinct high-throughput experiments to characterize samples of interest. There is a lack of methods to visualize these different genomic datasets in a versatile manner. In addition, genomic data analysis requires integrated visualization of experimental data along with constantly changing genomic annotation and statistical analyses.

**Results:**

We developed *GenomeGraphs*, as an add-on software package for the statistical programming environment R, to facilitate integrated visualization of genomic datasets. *GenomeGraphs *uses the *biomaRt *package to perform on-line annotation queries to Ensembl and translates these to gene/transcript structures in viewports of the *grid *graphics package. This allows genomic annotation to be plotted together with experimental data. *GenomeGraphs *can also be used to plot custom annotation tracks in combination with different experimental data types together in one plot using the same genomic coordinate system.

**Conclusion:**

*GenomeGraphs *is a flexible and extensible software package which can be used to visualize a multitude of genomic datasets within the statistical programming environment R.

## Background

Computational biologists are dealing with a growing range of genomic datasets, including microarray (e.g., mRNA, ChIP, SNP, CGH, and tiling-Chip) and ultra high-throughput sequencing (e.g., mRNA-Seq and ChIP-Seq) data. An increasing number of biological studies involve multiple, distinct, and high-throughput assays to characterize samples of interest. Novel and flexible visualization methods are needed to integrate these various data sources and combine them with annotation data from biological databases such as Ensembl [1].

Genome browsers such as the Ensembl Genome Browser [1], NCBI Entrez Map Viewer [2], and UCSC's Golden Path Genome Browser [3] enable upload and visualization of experimental data but have limited plotting options, do not provide data analysis capabilities of the displayed data, and are too far removed from the environment used to conduct statistical analysis. Other tools linking genome annotation to experimental data are mostly limited to a specific data type or rely on the Genome Browser's viewers for visualization. Statistical Viewer [4] for example facilitates interpretation of linkage and association data by providing a plug-in for data upload to the Ensembl Genome Browser.

The X:Map [5] genome annotation database and its companion software package *exonmap *enable integrated visualization of experimental data and genome annotation but it is specific to exon arrays and requires a local installation of the Ensembl database. It does not currently support visualization of multiple datasets and does not represent alternative splicing structures.

The main drawback of the tools described above is that they are not programmatically accessible and cannot be integrated into an analysis pipeline requiring batch processing. In addition, the required data upload step does not scale well for large and complex datasets.

The statistical programming environment R  along with the Bioconductor Project  provide a plethora of methods and tools to analyze and visualize data. The software package described in this paper, *GenomeGraphs*, builds on this functionality by providing an integrated API for direct visualization of data from a variety of sources. *GenomeGraphs *allows complex customization to facilitate a more complete integration and representation of genomic datasets.

## Implementation

### Graphic composition

#### Genomic dataset objects

We developed *GenomeGraphs *as an add-on package for the statistical programming environment R [6]. It utilizes the *S4 *class system and represents each genomic data type as a specific class. The root class *gdObject *provides basic functionality for display of data that can be mapped onto the genome (see Table 1). All data-type specific classes extend *gdObject *and corresponding display functionalities are built on top of this class. An example is the *GenericArray *class which represents gene expression microarray and arrayCGH data. This class takes a matrix of intensities as input which can easily be extracted from *ExpresionSet *objects as produced by the Bioconductor *affy *package. Another example is the *GeneRegion *class which represents strand-specific genes in a given genomic region. Quantitative genomic data, such as data from arrayCGH and tiling array experiments, frequently have associated segmented data. Segmented data are represented by the *Segmentation *class. Additional classes exist that represent ideograms, genomic axes, and legends. Regions of interest can be highlighted on the plot by using objects of the *RectangleOverlay *class. Once *gdObjects *are created, they can be visualized in one plot using the main plotting function, *gdPlot*.

**Table 1:** Overview of classes representing drawable genomic datasets

Class	Description
gdObject	the root class of the system, never directly instantiated
DisplayPars	class managing various plotting parameters
Gene	class representing a gene
GeneRegion	class defining a region of a chromosome, generally a set of genetic elements (genes)
Transcript	class defining a transcript
TranscriptRegion	class defining a region of a chromosome, generally a set of genetic elements (transcripts)
Ideogram	class representing an ideogram
Title	class to draw a title
Legend	class to draw a legend
GenomeAxis	class to draw an axis
AnnotationTrack	class used to represent custom annotation
Overlay	root class for overlays, never directly instantiated
RectangleOverlay	class to represent rectangular regions of interest
TextOverlay	class to draw text on plots
Segmentation	class to draw horizontal lines in various sets of data
GenericArray	class to draw data from microarrays.
ExonArray	class to draw data from exon microarrays.
GeneModel	class to draw custom gene models (intron-exon structures)
BaseTrack	class to draw arbitrary data at a given base
MappedRead	class to plot sequencing reads that are mapped to the genome

The main class, of which all other drawable classes are subclasses of, is the *gdObject *class. The *Gene*, *Transcript*, and *GeneRegion *classes are subclasses of *gdObject *and represent annotation data that are retrieved on-line from Ensembl upon creation of these objects. The third set of classes represent experimental data such as microarray and copy number data which can both be modeled using the *GenericArray *class. Probe-level exon array data can be represented by the *ExonArray *class and base-specific values such as nucleotide conservation scores among different species are represented by the *BaseTrack *class.

New technological developments to characterize cellular states may need novel representations. Classes representing these new data types can be easily added to *GenomeGraphs *and if the corresponding drawing methods are defined, the new data structures can be plotted using *gdPlot *along with data from existing classes.

#### Genome annotation retrieval from Ensembl using biomaRt

*GenomeGraphs *relies on the *biomaRt *package [7] to retrieve genomic annotation information on-line from Ensembl using BioMart web services [8]. The annotation information retrievable through *biomaRt *ranges from gene annotation, transcript isoforms to SNP data. This information can be retrieved from the most current release of Ensembl or from archived releases. By using *biomaRt*, there is no need for a local database installation of Ensembl, greatly facilitating the software installation procedure.

#### Custom genome annotation tracks

Ensembl contains annotation of a limited number of eukaryotic genomes. Any custom genome annotation can be visualized in *GenomeGraphs *by constructing instances of the *AnnotationTrack *class. For instance, genomic annotation encoded in GFF files can be easily used to create a custom *AnnotationTrack *object for visualization. To use the *AnnotationTrack *class, region start and end positions need to be given, as well as how these regions are to be grouped.

### Mapping of user data to genomic coordinates

*GenomeGraphs *is a visualization tool and as such does not provide mappings of user supplied data to the genome. Instances of the class *gdObject *take as input genomic coordinates provided by the user who is responsible for ensuring that these coordinates match the relevant genome annotation. To get the chromosomal coordinates of the data, users can either rely on the annotation provided by the platform which generated the data or on independently created mappings to the genome.

## Results

### Example I: arrayCGH and exon array data

In this first example, we illustrate how different genomic datasets can be visualized together in an integrated *GenomeGraphs *graphic. We use arrayCGH and Affymetrix exon array data and plot these together with genomic annotation from Ensembl.

We first load the *GenomeGraphs *package and one of its example datasets. This dataset contains copy number data and segmented copy number data, as well as exon array data for a small genomic region. Once the data are loaded, a *gdObject *is created for each data type, namely a *Segmentation *object containing the copy number segments, a *GenericArray *object containing the raw copy number data, an *Ideogram *object representing the relevant chromosome we are plotting, a *GenericArray *object containing the exon array data, and a *GenomeAxis *object for the genomic coordinate axis.

> library(GenomeGraphs)

> data('exampleData', package='GenomeGraphs')

> seg = makeSegmentation(value = segments,

   start = segStart, end = segEnd, dp = DisplayPars(color = 'dodgerblue2', lwd = 2, lty = 'dashed'))

> copyNumber = makeGenericArray(intensity = cn, probeStart = probestart,

   segmentation = seg, dp = DisplayPars(size = 3, color = 'seagreen', type="dot"))

> ideogram = makeIdeogram(chromosome = 3)

> expression = makeGenericArray(intensity = intensity, probeStart = exonProbePos,

      dp = DisplayPars(color='darkred', type='point'))

> genomeAxis = makeGenomeAxis(add53 = TRUE, add35 = TRUE)

In a next step, genomic annotation information is retrieved on-line from Ensembl using the *biomaRt *package. We first connect to the Ensembl BioMart database and select the human (*hsapiens*) dataset. Then, we retrieve gene structures on the forward and reverse strands of the region we want to visualize.

> minbase = 180292097

> maxbase = 180492096

> mart = useMart('ensembl', dataset='hsapiens_gene_ensembl')

> genesplus = makeGeneRegion(start = minbase, end = maxbase, strand = '+', chromosome = '3', biomart = mart)

> genesmin = makeGeneRegion(start = minbase, end = maxbase, strand = '-', chromosome = '3', biomart = mart)

**Figure 1:**
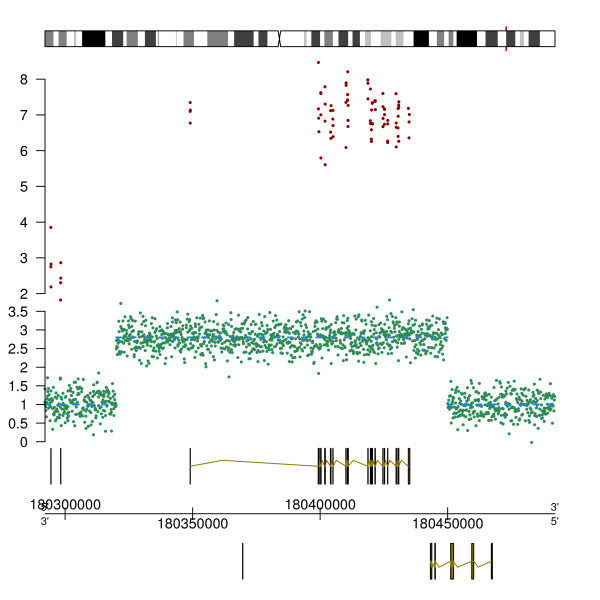
**ArrayCGH and exon array data**. The first track in this figure shows an ideogram of the human chromosome 3. The red marker highlights the plotted genomic region. The second track shows exon array data, where each data point corresponds to a probe measuring the expression level of an exon. The third track displays copy number data in green and segmented copy number data with dashed blue lines. Note the amplification which can be seen in both the copy number and exon array tracks, suggesting that the amplification event results in higher expression levels of the gene in this region. The bottom track shows the gene annotation data from Ensembl.

In a last step, the *gdPlot *function is called to plot instances of *gdObject *that were created above. The objects are given to *gdPlot *as a list and the order in the list will determine the plotting order from top to bottom. A minimum and maximum base position are also given as arguments to restrict the visualization to this particular genomic region. The plot produced from this example is shown in Figure 1.

> gdPlot(list(ideogram, expression, copyNumber, genesplus, genomeAxis, genesmin), minBase = minbase, maxBase = maxbase)

### Example II: Transcript isoforms and exon array data

**Figure 2:**
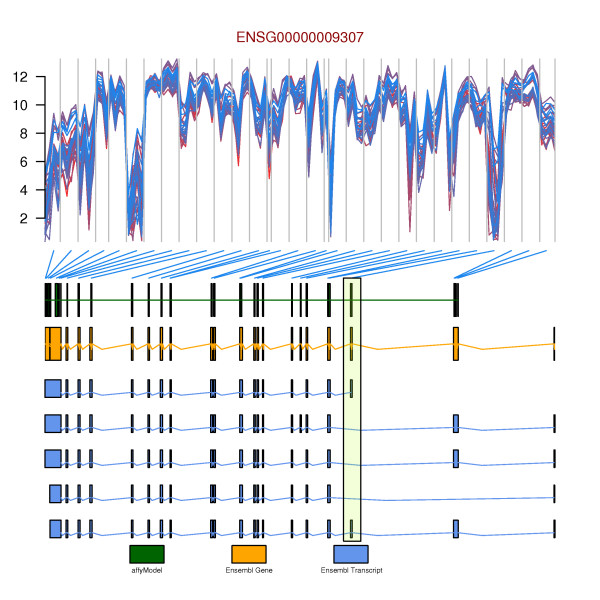
**Transcript isoforms and exon array data**. Probe-level exon array data is plotted in the top graphic. The data of the exon array is intentionally not plotted on the exact chromosomal location of the probes in order to clearly visualize alternative splicing events. Each line in the top track represents a different sample. Usually, there are four probes per exon on the Affymetrix GeneChip^® ^Human Exon 1.0 ST platform, vertical gray lines group these four probes belonging to the same exon together. The blue connecting lines map these exons to gene models as defined by Affymetrix (green) and Ensembl (orange). Transcript isoforms known for this gene are plotted in dark blue. The region highlighted in the plot by an *RectangleOverlay *object shows the exon that is not expressed in the samples. One can see that this is a known alternatively spliced exon as annotated by Ensembl.

In a second example, we show how probe-level exon array data from the Affymetrix GeneChip^® ^Human Exon 1.0 ST platform (data available from ), can be plotted along with gene models from Affymetrix as well as gene and transcript annotation from Ensembl. The data of the exon array are not plotted at the exact chromosomal location of the probes in order to clearly visualize alternative splicing events. Most of the exons are represented on the Human Exon 1.0 ST platform by four probes. The location of these four probes are equally spaced in the data plots. Each exon is separated by a vertical line and the exons are linked to their genomic location by connecting lines. This visualization makes it easy to relate alternative exon usage, as observed in the exon array data, to known alternative transcript isoforms in Ensembl (Figure 2). The region highlighted in the plot shows the exon that is not expressed in the samples. To generate this plot, we first create the different subclasses of *gdObject*, namely: *Title*, *ExonArray*, *Gene*, *Transcript*, and *Legend *objects. In addition, we make a custom annotation track using the *AnnotationTrack *class.

> data('unrData', package='GenomeGraphs')

> title = makeTitle(text ='ENSG00000009307', color = 'darkred')

> col = colorRampPalette(c('firebrick2','dodgerblue2'))(length(unrData[1,]))

> exon = makeExonArray(intensity = unrData, probeStart = unrPositions[,3], probeEnd = unrPositions[,4],

   probeId = as.character(unrPositions[,1), nProbes = unrNProbes,

   dp = DisplayPars(color = col, mapColor = 'dodgerblue2'), displayProbesets = FALSE)

> affyModel <- makeAnnotationTrack(start = unrPositions[,3], end = unrPositions[,4],

         feature = "gene_model", group = "ENSG00000009307",

         dp = DisplayPars(gene_model = "darkblue"))

> gene = makeGene(id = 'ENSG00000009307', biomart = mart)

> transcript = makeTranscript(id ='ENSG00000009307', biomart = mart)

>legend = makeLegend(text = c('affyModel','Ensembl Gene', 'Ensembl Transcript'),

      fill = c('darkgreen','orange','cornflowerblue'), cex = 0.5)

In a second step, we use the *RectangleOverlay *class to create a highlighted region followed by the *gdPlot *function to produce the integrated plot.

> rOverlay = makeRectangleOverlay(start = 115085100, end = 115086500, region = c(3,5),

   dp = DisplayPars(alpha = .2, fill = "olivedrab1"))

> gdPlot(list(title, exon, affyModel, gene, transcript, legend), minBase = 115061061, maxBase = 115102147, overlay = rOverlay)

The plot generated in this second example is shown in Figure 2.

### Example III: Short read sequencing and tiling array data

In the final example, we show how complex and diverse sets of data can be integrated to facilitate joint analysis and draw biological conclusions by presenting data from various published datasets on yeast. First, we construct a list where each *gdObject *represents either annotation or a publicly available dataset. We have plotted data from Ensembl, an Illumina sequencing dataset [9], Affymetrix tiling array data [10], nucleosome position data [11], and conservation data across 7 related species [12].

> data("seqDataEx", package = "GenomeGraphs")

> str = seqDataEx$david [,"strand"] == 1

> biomart = useMart("ensembl", "scerevisiae_gene_ensembl")

> pList = list("-" = makeGeneRegion(chromosome = "IV", start = 1300000, end = 1310000,

      strand = "-", biomart = biomart,

      dp = DisplayPars(plotId = TRUE, idRotation = 0, cex = .5)),

   makeGenomeAxis(dp = DisplayPars(byValue = 1e3, size = 3)),

   "+" = makeGeneRegion(chromosome = "IV", start = 1300000, end = 1310000,

      strand = "+", biomart = biomart,

      dp = DisplayPars(plotId = TRUE, idRotation = 0, cex = .5)),

   "Nagalakshmi" = makeBaseTrack(base = seqDataEx$snyder [, "location"], value = seqDataEx$snyder [, "counts"],

         dp = DisplayPars(lwd = .3, color = "darkblue", ylim = c(0,300))),

   "David +" = makeGenericArray(probeStart = seqDataEx$david [str, "location"],

         intensity = seqDataEx$david [str, "expr", drop = FALSE],

         dp = DisplayPars(pointSize = .5)),

   "David -" = makeGenericArray(probeStart = seqDataEx$david [!str, "location"],

         intensity = seqDataEx$david [!str, "expr", drop = FALSE],

         dp = DisplayPars(color = "darkgreen", pointSize = .5)),

   "Lee" = makeBaseTrack(base = seqDataEx$nislow [, "location"],

      value = seqDataEx$nislow [, "evalue"], dp = DisplayPars(color="grey", lwd = .25)),

   "Conservation" = makeBaseTrack(base = seqDataEx$conservation [, "location"],

         value = seqDataEx$conservation [, "score"],

         dp = DisplayPars(color="gold4", lwd = .25)))

Having constructed the list of elements we wish to plot, we now set up an overlay, using the *RectangleOverlay *class, to highlight a region of interest. Finally, we plot the result using *gdPlot*. Although configuring and designing the initial plot may seem laborious, once we have this basic structure we can easily produce plots for all regions of interest.

> rOverlay = makeRectangleOverlay(start = 1302105, end = 1302190, region = c(4,8), dp = DisplayPars(alpha = .2))

> gdPlot(pList, minBase = 1301500, maxBase = 1302500, overlay = rOverlay)

**Figure 3:**
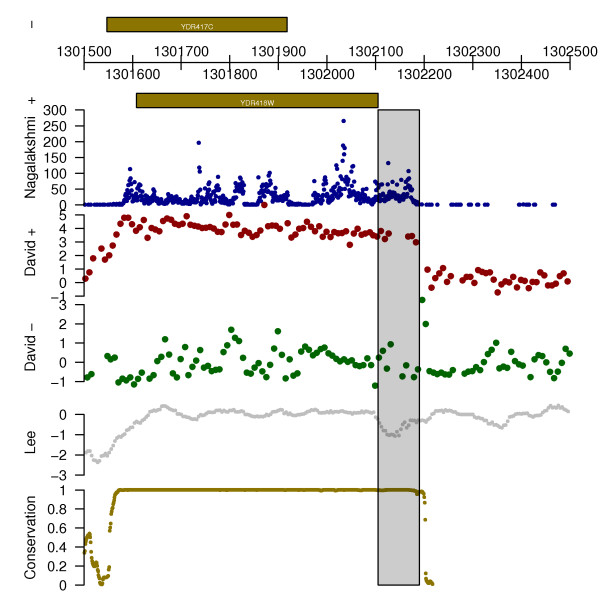
**Short read sequencing and tiling array data**. Data plotted are Illumina sequencing data from Nagalakshmi et al. [9], tiling array data from David et al. [10], nucleosome data from Lee et al. [11], and conservation track data from Siepel et al. [12]. The semi-transparent box highlights a possible annotation error in SGD, as suggested by the occurrence of the transcript in multiple separate datasets. In addition, the conservation track data demonstrate corroborating evidence for the possibility of a longer gene.

The plot produced in this third example is shown in Figure 3.

## Conclusion

*GenomeGraphs *is a versatile and extensible visualization package in R, which is well suited to create integrated displays of diverse experimental datasets and genomic annotation information. By using the *biomaRt *package, annotation information is retrieved directly from Ensembl and there is no need to install and maintain annotation databases locally. Custom annotation tracks can also be created by using the *AnnotationTrack *class. Finally, *GenomeGraphs *provides the user with tight integration into R providing immediate access to a wealth of statistical methods.

The software package comes with a vignette which is an executable document that demonstrates how to use the package. The examples described in this paper are also included in the vignette and can be executed after installation of the package. More complex features are also demonstrated in the vignette. Future versions of the package will include more flexibility in terms of plotting parameters and plotting novel features such as visualizing SNP information as annotated by Ensembl and stacked sequencing read representations.

## Availability and requirements

*GenomeGraphs *is an open source software package under the *Artistic-2.0 *license and has been contributed to the Bioconductor Project. The software and source code are available for download from . This document was produced using R-2.8.0 and *GenomeGraphs *version 1.2.0 available at the following URL: . The package has been tested and run on OS X, Windows, and a variety of Linux systems. *GenomeGraphs *depends on the following software packages *XML*, *RCurl*, and *biomaRt*, which can be downloaded from Bioconductor or installed from R using the  script. The versatility of *GenomeGraphs *visualization relies on the powerful R plotting package *grid *[13]. Each *gdObject *is plotted in an individual *viewPort *from the *grid *package. *Grid *is typically installed together with the base installation of *R*.

## Authors' contributions

SD and JB developed the software package. PS and SD provided scientific advice and the resources to develop the software.
